# Work-Related De Quervain’s Tensosynovitis (DQT): The Diagnosis Dilemma

**DOI:** 10.7759/cureus.33458

**Published:** 2023-01-06

**Authors:** Faisal Al Badri

**Affiliations:** 1 Occupational Medicine, Armed Forces Medical Services, Muscat, OMN

**Keywords:** exposure assessment, occupational, work-related, ergonomic hazards, de quervain’s tenosynovitis

## Abstract

A female presented with right wrist pain for nine months. The diagnosis was De Quervain’s tenosynovitis; her condition was initially managed conservatively, but later she needed surgery due to the lack of improvement. No workplace intervention had been implemented because her hand surgeons did not consider her occupational exposures. It was later discovered that there was significant exposure to ergonomic hazards at her workplace. She received accommodations in her workplace that led to reduction of exposure to ergonomic hazards and the subsequent dramatic improvement in her condition. This case report indicates that some orthopedists do not appreciate the role of occupational exposure to ergonomic hazards in the pathogenesis of similar conditions. Occupational medicine specialists and orthopedists should, therefore, communicate with each other to reach a consensus regarding the association between occupational exposures to ergonomic hazards and work-related upper limb disorders (WRULDs).

## Introduction

Work-related upper limb disorders (WRULDs) have been increasingly recognized as a major and costly health issue in the workplace [1-2]. Despite the fact that De Quervain’s tenosynovitis (DQT) has the lowest incidence among all WRULDs [3], it has been estimated that DQT, regardless of its etiology, results in the loss of two million working days per year in developed countries [4]. Furthermore, depending on the population studied and the diagnostic criteria, the prevalence of DQT ranges between 0.7% and 36% in working populations [3, 5-12]. In addition, incidence rates of 0.6 per 1000 person-years in men and 2.8 per 1000 person-years in women were found in a study involving a very large population of young US military personnel [13]. The same study also found that women were three times more likely than men to develop DQT. This statistics indicate that DQT is not an uncommon disorder in the working population and, as a result, occupational medicine specialists need to consider it in their differential diagnosis when they evaluate patients with wrist pain. Similarly, orthopedists and hand surgeons need to recognize the importance of workplace exposure to ergonomic hazards in the pathogenesis of DQT and provide recommendations regarding ways to minimize the risk at work. Also, they need to remember that DQT is a reportable condition in many countries, including South Africa as per Circular Instruction No. 180 [14].

## Case presentation

The patient was a 48-year-old-woman who was referred to the Occupational Medicine Clinic on 6th June 2017 by her employer through an occupational therapist for an evaluation of the relationship between her condition and her work. She had complained of progressive intermittent right wrist pain for the past 9 months. It started at the base of her right thumb and radiated to the tip of the finger. It worsened with any movement of the thumb, especially during writing. The pain had been associated with numbness and tingling of the right thumb for the last few weeks. There was no history of trauma or rheumatological diseases. There were no night symptoms or swelling. There was also no swelling, stiffness, or joint deformity of any other joint. She is right-handed.

She was seen twice by hand surgeons and was diagnosed with DQT. She received two local steroid injections and was advised to wear a splint most of the time. She was prescribed paracetamol 1 g and tramadol hydrochloride 1 g PRN as well. However, there was no significant improvement according to the patient. Her past medical history was unremarkable. There was no significant exposure to ergonomic hazards at home or during her leisure time.

She started working at a large tertiary hospital in 1989 as an assistant nurse, and she worked in that role for 20 years before she became a staff nurse in 2004. She moved to a private hospital in 2010. Her tasks in these roles were basic nursing care duties including washing and feeding patients. In 2013, she started working in a primary health care clinic. Initially, her tasks were triaging patients and measuring vital signs. In 2015, she took over the pharmacy duties as well because the pharmacy nurse retired. Since then, she has been performing other tasks such as writing on the medication’s small bags, opening and closing zip-lock bags, and stapling packets. While accomplishing these tasks, she performs frequent repetitive movements such as thumb opposition, flexion and extension of the thumb and wrist as well as ulnar and radial deviation of the wrist. These tasks require a high degree of manual dexterity.

The general examination yielded non-significant findings except for the fact that she was overweight. The right wrist joint examination revealed swelling with mild tenderness, but there was no warmth, erythema, or finger deformity. Finkelstein's test was strongly positive, but Phalen’s test, Tinel’s test and Grind test were negative. There was full range of movement but with significant tenderness. Sensation, motor examinations, and crude grip strength were normal. Examinations of the left wrist and other large joints were unremarkable. Finally, she underwent a functional capacity assessment on March 2017 according to a request made by her employer, and the results showed a decrease in grip strength of the right hand during cylindrical, pencil grip, and pinch grasp tests.

Investigations

The patient was clinically diagnosed with DQT by a hand surgeon based on her history and the clinical examinations. There was no special investigation needed to reach the diagnosis. However, the hand surgeons did not investigate the contribution of occupational risk factors to her DQT. Therefore, we conducted a workplace visit to evaluate the ergonomic risk factors at her workplace using the Assessment of Repetitive Tasks of the upper limbs tool (ART tool) [15]. The left-hand score was 17, and the right-hand score was 23. A score between 12 and 21 indicates a medium exposure level, with attention needed to minimize the exposure. A score of 22 or higher indicates a high exposure level, and attention is urgently needed to minimize the exposure. Finally, a functional capacity assessment was performed, and marked impairment of her dominant hand and difficulty in performing her tasks at the workplace were observed.

Differential diagnosis

The differential diagnosis included DQT, osteoarthritis of the trapeziometacarpal joint, and gout. However, this case is an example of classic work-related DQT. Firstly, the diagnosis of DQT was made by a medical practitioner and was supported by the typical clinical history and examinations, specifically the strongly positive Finkelstein's test. Secondly, although the patient had important risk factors for DQT such as age and gender, but her occupational exposure to ergonomic hazards contributed substantially to her condition, as confirmed by the ART tool. Furthermore, the chronological relationship between the condition and the exposure makes the diagnosis of work relatedness highly likely. Finally, the functional capacity assessment showed marked impairment of her dominant hand and difficulty in performing her tasks at her workplace.

It was the clinical director at her workplace who suspected a possible relationship between her condition and her work. Theoretically, we would have missed this patient if she had not been referred for further evaluation by her employer. 

Treatment

The patient was initially managed by pharmacological treatments, including oral non-steroidal anti-inflammatory drugs (NSAIDs) and multiple local steroid injections, and a non-pharmacological treatment, namely splinting of the affected hand. At our clinic, the first medical report was submitted to the Compensation Fund and the employer was notified about the condition of the employee and the recommendation has been made for the employer to accommodate the patient in another area where there is lower ergonomic risk.

Outcome

Two months after her first visit to our clinic, the patient underwent a surgical intervention on the hand. According to the surgical notes, the first compartment was identified and released as well as both tendons, the abductor pollicis longus (APL), and the extensor pollicis brevis (EPB). She was given a sick leave for 6 weeks and asked to resume her duties. Six months after the surgery, it was found that the patient had not been accommodated despite our recommendations, which resulted in non-improvement of her condition. Therefore, we communicated again with the employer asking for accommodation of the patient in another area where she does not have to perform repetitive movements of the upper limbs. Six months later, the patient was followed up at her workplace and found that she had been accommodated 4 months earlier and had no/less exposure to repetitive movements, which had resulted in a dramatic improvement in her symptoms.

## Discussion

The strengths and limitations of the management of this case

There were strengths in the management of this case. Firstly, the supervisor of the patient is a medical doctor and she recognized the possibility of the association of the patient’s condition and her work environment. Therefore, she referred the patient to our clinic for further evaluation. Furthermore, the ART tool was comprehensive and easy to use. Regarding the limitations of the management of this case, the hand surgeons who initially treated the patient did not consider the possible association between DQT and the patient’s work environment. Therefore, the patient was not initially informed about the possible association and was not educated about the precautions she should take to improve her condition. Secondly, it was difficult for the patient to wear the splint while performing her routine tasks. Furthermore, the patient was not initially accommodated despite the clear recommendations because of a staff shortage in her workplace. The patient’s condition, therefore, did not initially improve, and she had to undergo a surgical intervention. However, the patient was later accommodated after multiple communications with the employer. One of the reasons for this delay in the workplace accommodation could be attributed to the long intervals between Occupational Medicine OPD Clinic visits. We could have intervened earlier if the patient was seen in a more timely manner.

Diagnosis of De Quervain’s tenosynovitis

De Quervain’s tenosynovitis affects the APL and the EPB and involves thickening of these tendons as well as the thickening and swelling of the extensor retinaculum, the first extensor compartment of the wrist at the styloid process of the radius, through which the two tendons pass [13, 16-18]. Moreover, DQT is characterized by pain in the region of the radial styloid that is aggravated by ulnar deviation of the wrist and/or the thumb.

Examinations may reveal tenderness and swelling of the radial styloid region. The widely excepted test, Finkelstein’s test, is considered to be the pathognomonic sign of DQT [19]. Because of the fact that X-ray of the wrist is usually normal, and ultrasound is often not needed despite its ability to show positive diagnostic characteristics [17], the diagnosis is usually based on the clinical history and examinations.

A multidisciplinary team, Harrington et al. [18], have proposed a consensus on the diagnostic criteria for DQT. The criteria they defined are “pain which is centred over the radial styloid and tender swelling of first extensor compartment and either pain reproduced by resisted thumb extension or positive Finkelstein’s test.” However, we should consider other conditions that may present with similar symptoms and signs which include osteoarthritis of the trapeziometacarpal joint, intersection syndrome, ganglia, radial sensory nerve entrapment in the forearm and gout [17].

Work relatedness

In addition to the risk factors of age greater than 40 years and female gender [13], it is believed that DQT results from prolonged repetitive and forceful manual work [20]. The frequency of the repetitive hand movements was arbitrarily estimated to be >20 movements per minute [21]. However, despite the recognition of the association between workplace exposure to ergonomic hazards and the aetiology of DQT [3, 5-10, 12, 22], the role of workplace exposure to ergonomic hazards in the etiology of DQT has been challenged in the past by Stahl et al. [23]. In their review, Stahl et al. identified 47 papers that discussed the association between occupational exposure and the pathogenesis of DQT. Forty-one articles supported the hypothesis of a causal relationship, while six articles refuted it. Interestingly, despite the significant association they reported between DQT and work-related factors, (OR: 2.89; 95 percent CI: 1.4 to 5.97; p = 0.004), they concluded that there is insufficient evidence to confirm a causal association between DQT and work-related factors. They reached this conclusion by applying the Bradford Hill criteria. Furthermore, Dunn et al. has supported Stahl et al. in their conclusion and excluded any possible causal association between DQT and work-related factors [24].

Interestingly, it seems that the background of the first author of these articles played a very important role in the conclusions drawn in the papers. It was found that surgeons are less likely to consider occupational risk factors when they evaluate DQT patients [23]. This tendency to underestimate work relatedness was clear in the opinion of the British Orthopaedic Association (BOA). While the British Health and Safety Executive (HSE) states that physically demanding and repetitive work put workers at increased risk of developing DQT, the opinion of the BOA is that “De Quervain's disease is rarely caused by occupation” [25-27].

The exclusion of a causal association between DQT and work-related factors should be considered with caution as there is insufficient scientific evidence to support it. Firstly, there is a lack of precise definitions of repetitive movements and the intensity of forceful manual work, and the definitions of DQT and work exposure vary in the literature [23, 28]. Secondly, well-designed longitudinal studies that evaluate this association are very scant. Thirdly, using the Bradford Hill criteria solely to exclude this association should be looked at prudently as their practicability in determining work relatedness has been questioned [29-30]. These criteria increase the confidence in the existence of a causal relationship when they are met. However, they were not intended to exclude a causal relationship if they are not met [31]. Furthermore, despite the fact that some populations are more prone genetically or anatomically to developing DQT, we should question whether this predisposition is sufficient to cause the disease. What triggers the pathological changes in the first compartment and the associated tendons? By using counterfactual thinking, if predisposed workers are not exposed to the work-related factors, well they develop DQT? In my opinion, in the working population, there is a strong correlation between work-related factors and the development of DQT, but more studies from multidisciplinary teams that include occupational medicine physicians and hand surgeons are needed to support or refute causal relationships in similar multifactorial conditions. Since most of the cases of DQT are managed by surgeons, I think many work-related cases are missed each year, leading to underreporting. Furthermore, the prognosis of such cases might be affected because the patients continue to perform physically demanding and repetitive work if the link between the etiology of the disease and the work environment has not been considered. Therefore, the presence of a well-trained occupational health nurse or an occupational health practitioner in the workplace is essential to integrate the input from hand surgeons, occupational medicine specialists, and employers into a well-defined management plan and to monitor the prognosis of the affected worker.

## Conclusions

Despite early surgical intervention, the status of workers with WRULDs may worsen or fail to improve if they are not accommodated and moved to areas with no/less exposure to ergonomic hazards. Simple and validated risk assessment tools that assist in the evaluation of the exposure of upper limbs to ergonomic hazards are available, and some do not need previous ergonomic skills. Furthermore, occupational medicine specialists, orthopedists, and hand surgeons need to reach a consensus regarding the association between occupational exposure and WRULDs. Finally, supervisors and health and safety representatives with adequate knowledge of ergonomic hazards at the workplace may play a significant role in protecting and managing their employees.

## References

[1] National Research Council (US) and Institute of Medicine (US) (2001). Musculoskeletal Disorders and the Workplace: Low Back and Upper Extremities.

[2] Punnett L, Wegman DH (2004). Work-related musculoskeletal disorders: the epidemiologic evidence and the debate. J Electromyogr Kinesiol.

[3] Roquelaure Y, Ha C, Leclerc A (2006). Epidemiologic surveillance of upper-extremity musculoskeletal disorders in the working population. Arthritis Rheum.

[4] Liebers F, Caffier G (2009). Job Specific Sickness Absence Due to Musculoskeletal Disorders in Germany.

[5] Bystrom S, Hall C, Welander T, Kilbom A (1995). Clinical disorders and pressure-pain threshold of the forearm and hand among automobile assembly line workers. J Hand Surg Br.

[6] Kurppa K, Viikari-Juntura E, Kuosma E, Huuskonen M, Kivi P (1991). Incidence of tenosynovitis or peritendinitis and epicondylitis in a meat-processing factory. Scand J Work Environ Health.

[7] Luopajarvi T, Kuorinka I, Virolainen M, Holmberg M (1979). Prevalence of tenosynovitis and other injuries of the upper extremities in repetitive work. Scand J Work Environ Health.

[8] McCormack RRJ, Inman RD, Wells A, Berntsen C, Imbus HR (1990). Prevalence of tendinitis and related disorders of the upper extremity in a manufacturing workforce. J Rheumatol.

[9] Gold JE, d'Errico A, Katz JN, Gore R, Punnett L (2009). Specific and non-specific upper extremity musculoskeletal disorder syndromes in automobile manufacturing workers. Am J Ind Med.

[10] Viikari-Juntura E (1983). Neck and upper limb disorders among slaughterhouse workers. An epidemiologic and clinical study. Scand J Work Environ Health.

[11] Walker-Bone K, Palmer KT, Reading I, Coggon D, Cooper C (2004). Prevalence and impact of musculoskeletal disorders of the upper limb in the general population. Arthritis Rheum.

[12] Petit Le Manac’h A, Roquelaure Y, Ha C (2011). Risk factors for de Quervain’s disease in a French working population. Scand J Work Environ Health.

[13] Wolf JM, Sturdivant RX, Owens BD (2009). Incidence of de Quervain's tenosynovitis in a young, active population. J Hand Surg Am.

[14] Department of Labour (2004). Department of Labour. Circular Instruction 180 Regarding the Compensation of Work-Related Upper Limb Disorders (WRULDs).

[15] Ferreira J, Gray M, Hunter L, Birtles M, Riley D (2009). Development of An Assessment Tool for Repetitive Tasks of the Upper Limbs (ART).

[16] Ilyas AM, Ast M, Schaffer AA, Thoder J (2007). De quervain tenosynovitis of the wrist. J Am Acad Orthop Surg.

[17] Aggarwal R, Ring D (2018). de Quervain tendinopathy. https://www.uptodate.com/contents/de-quervain-tendinopathy?search=DeQuervain’sTensosynovitis&source=search_result&selectedTitle=1~150&usage_type=default&display_rank=1.

[18] Harrington JM, Carter JT, Birrell L, Gompertz D (1998). Surveillance case definitions for work related upper limb pain syndromes. Occup Environ Med.

[19] Dawson C, Mudgal CS (2010). Staged description of the Finkelstein test. J Hand Surg Am.

[20] Stahl S, Vida D, Meisner C, Stahl AS, Schaller HE, Held M (2015). Work related etiology of de Quervain's tenosynovitis: a case-control study with prospectively collected data. BMC Musculoskelet Disord.

[21] Hammer A (1934). Tenosynovitis. Med Rec.

[22] Thompson AR, Plewes LW, Shaw EG (1951). Peritendinitis crepitans and simple tenosynovitis; a clinical study of 544 cases in industry. Br J Ind Med.

[23] Stahl S, Vida D, Meisner C, Lotter O, Rothenberger J, Schaller HE, Stahl AS (2013). Systematic review and meta-analysis on the work-related cause of de Quervain tenosynovitis: a critical appraisal of its recognition as an occupational disease. Plast Reconstr Surg.

[24] Dunn JC, Polmear MM, Nesti LJ (2019). Dispelling the myth of work-related de Quervain's tenosynovitis. J Wrist Surg.

[25] (2018). The Health and Safety Executive. Occupational diseases - Reportable diseases [Internet] [cited May 20]. http://www.hse.gov.uk/riddor/occupational-diseases.htm.

[26] Silman AJ, Newman J (1996). A review of diagnostic criteria for work related upper limb disorders (WRULD). Arthritis Rheum Counc Epidemiol Res Unit. A Review of Diagnostic Criteria for Work Related Upper Limb Disorders (WRULD).

[27] Barton NJ, Hooper G, Noble J, Steel WM (1992). Occupational causes of disorders in the upper limb. BMJ.

[28] Miranda H, Viikari-Juntura E, Heistaro S, Heliövaara M, Riihimäki H (2005). A population study on differences in the determinants of a specific shoulder disorder versus nonspecific shoulder pain without clinical findings. Am J Epidemiol.

[29] Rothman KJ, Greenland S (2005). Causation and causal inference in epidemiology. Am J Public Health.

[30] Frank JW, Frcp C, Maetzel A (1998). Frank JW, Frcp C, Maetzel A. Determining what constitutes an occupational disorder: is the horse already well out of the barn door?. Authors : [Internet.

[31] Kundi M (2006). Causality and the interpretation of epidemiologic evidence. Environ Health Perspect.

